# Solar Photooxygenations for the Manufacturing of Fine Chemicals—Technologies and Applications

**DOI:** 10.3390/molecules26061685

**Published:** 2021-03-17

**Authors:** Jayson S. Wau, Mark J. Robertson, Michael Oelgemöller

**Affiliations:** 1 College of Science and Engineering, James Cook University, Townsville, QLD 4811, Australia; jayson.wau@my.jcu.edu.au (J.S.W.); mark.robertson@jcu.edu.au (M.J.R.); 2 Department of Organic and Macromolecular Chemistry, Ghent University, Krijgslaan 281 S4, 9000 Gent, Belgium

**Keywords:** solar chemistry, photooxygenations, solar reactor, chemical manufacturing

## Abstract

Photooxygenation reactions involving singlet oxygen (^1^O_2_) are utilized industrially as a mild and sustainable access to oxygenated products. Due to the usage of organic dyes as photosensitizers, these transformations can be successfully conducted using natural sunlight. Modern solar chemical reactors enable outdoor operations on the demonstration (multigram) to technical (multikilogram) scales and have subsequently been employed for the manufacturing of fine chemicals such as fragrances or biologically active compounds. This review will highlight examples of solar photooxygenations for the manufacturing of industrially relevant target compounds and will discuss current challenges and opportunities of this sustainable methodology.

## 1. Introduction

Photochemistry has recently seen a remarkable renaissance in synthetic organic chemistry [1,2,3,4]. This trend was sparked by the development of new photochemical transformations [5,6,7,8] as well as advances in photoreactor and light technologies [9,10,11,12,13]. However, the large-scale realization of photochemical manufacturing processes is hindered by their quantum yields and the significant installation and maintenance costs of artificial light sources [14,15]. As a result, industrial photochemistry is restricted to low-volume but high-value fine chemicals (such as fragrances, flavors or vitamins), or bulk but low-value chemicals (such as haloalkanes, oximes, sulfonyl chlorides and sulfonic acids) [14,15,16,17,18,19]. Natural sunlight is regarded as an alternative and sustainable energy source for a range of photochemical transformations [20,21,22,23]. Up until the early 20th century, photochemical experiments were routinely performed by placing sealed flasks and tubes in direct sunlight [24,25,26,27]. Although this ‘flask in the sun’ approach is still followed today [28,29], the small scales and prolonged exposure times result in very low productivities of the desired products. Recently, sunlight collectors and concentrators that were initially developed for energy generation [30,31,32,33] have been modified for solar photochemical experimentation [34]. Technical-scale solar degradation and detoxification processes have subsequently been successfully realized using these technologies [35,36,37,38]. In contrast, however, preparative solar chemistry for the manufacturing of chemicals is still comparably rare [39,40,41,42,43,44,45,46,47]. The main hurdles for a widespread implementation of solar synthesis are the discontinuous availability and the low UV content (<400 nm) of just 3–5% of natural sunlight [48]. Sunlight also consists largely of direct and diffuse radiation and their ratios vary with the location and weather [49]. Solar manufacturing may thus be ideally performed in the visible range (400–700 nm: 42–43% of solar spectrum [48]) for small annual production targets (<100 tons/year) that do not require continuous, year-round operation under optimal solar light conditions. Photooxygenations with singlet oxygen (^1^O_2_) combine catalytic amounts of a dye sensitizer with visible light and yield a variety of oxygenated products [50,51,52]. Photooxygenations are also performed industrially to produce low-volume fine chemicals such as fragrances, flavors or pharmaceuticals [53,54,55]. These features make photooxygenations ideal candidates for solar chemical manufacturing processes.

## 2. Solar Reactors for Synthetic Applications

**Figure 1:**
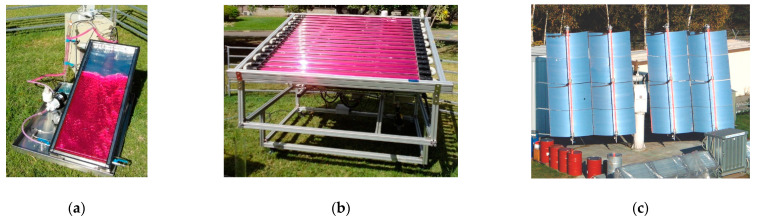
(**a**) Circulating flatbed reactor with reflective back and external cooling at James Cook University in Townsville, Australia. (**b**) CPC reactor with a 2 m^2^ aperture at James Cook University in Townsville, Australia. (**c**) Former PROPHIS loop at the German Aerospace Center (DLR) in Cologne-Porz, Germany (modified from [60]).

Solar reactors are classified based on their sun concentration factor (CF) and range from non- to highly concentrating systems. Non- and low concentrating reactors are characterized by large apertures that enable them to harvest direct and diffuse radiation. The simplest non-concentrating devices are flatbed reactors with large surface areas, thin bodies, and operation volumes of up to 30 L (Figure 1a) [56]. These fixed reactors are best tilted to the latitude of the location for optimal harvest of sunlight. Improved models operate in circulation mode with an external heat exchanger. The compound parabolic collector (CPC) uses a ‘round W’-shaped polished aluminum reflector that is tilted to the local latitude and directs all available radiation onto a central received tube (Figure 1b) [57,58]. These advanced systems have a concentration factor of one sun (or slightly above) and most commonly operate in circulating batch mode on volumes of up to 100 L. Several reactors can also be connected in series to form larger units. Parabolic trough reactors utilize large reflectors that focus only direct sunlight onto a reaction tube in their focal line and can reach concentration factors of 20–150 suns [32]. More modern reactors such as the former PROPHIS loop (PaRabolic trough-facility for Organic PHotochemical synthesis, Figure 1c) operate on volumes up to 150 L and track the movement of the sun, either horizontally or three-dimensionally (horizontally and vertically) [59].

While solar dish concentrators can provide very high concentration factors of up to 5000 suns, these sophisticated devices are rarely used for preparative solar photochemical applications. Since the receiver in the focal point easily reaches extreme temperature ranges, dish reactors are instead advantageous for thermochemical processes [33]. Likewise, solar furnaces can reach extreme concentration factors of 5000–20,000 suns by reflecting sunlight via a heliostat or heliostat field onto a concentrator, which then focuses the sun’s beam onto the experimental setup. The naturally extreme heat generation and the costly installation have limited the application of solar furnaces mainly to material sciences and solar thermal research activities [61,62,63].

## 3. Photooxygenations in Organic Synthesis

**Scheme 1:**
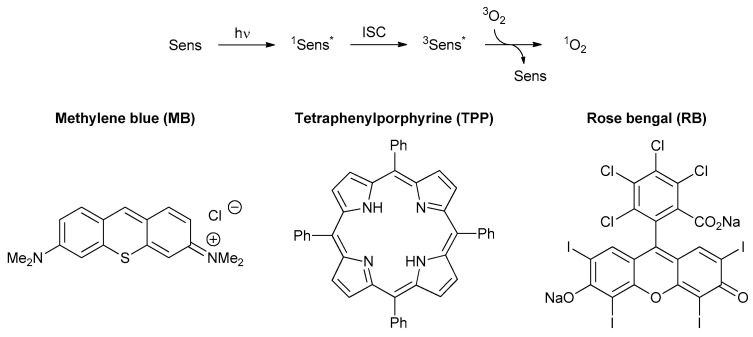
Photosensitized generation of singlet oxygen and common photosensitizers.

Photooxygenations, also known as Type II photosensitized oxidations [64], utilize singlet oxygen (^1^O_2_), which is generated from ground-state triplet oxygen (^3^O_2_) via photosensitization (Scheme 1) [65]. The sensitizer (Sens) initially absorbs light and undergoes intersystem crossing (ISC) to its triplet excited state (^3^Sens*). Subsequent energy transfer to ground state oxygen produces singlet oxygen. The dissolved oxygen concentration in most organic solvents is low [66,67] and thus, fine streams of oxygen or compressed air are constantly passed through the reaction media. Likewise, the lifetime of singlet oxygen critically depends on the reaction medium and is highest in halogenated solvents [67]. Due to the environmental hazards linked to these solvents, many industrial and solar photooxygenation processes are conducted in less harmful alcoholic solvents instead [68]. A series of photosensitizers with large absorption coefficients within the visible spectrum, suitable solubilities in a range of organic solvents, long triplet lifetimes and high quantum yields for ^1^O_2_ generation have now become available [69,70]. The most common photosensitizers methylene blue, tetraphenylporphyrin and rose bengal (as disodium salt) are shown in Scheme 1. For easy recovery and reuse, photosensitizers have also been immobilized on an inert solid support [71,72]. Photooxygenations have furthermore been successfully performed under continuous-flow conditions [73]. Thermal oxygenation processes involving ^1^O_2_ have likewise been developed but are at present less important in organic synthesis [74].

**Scheme 2:**
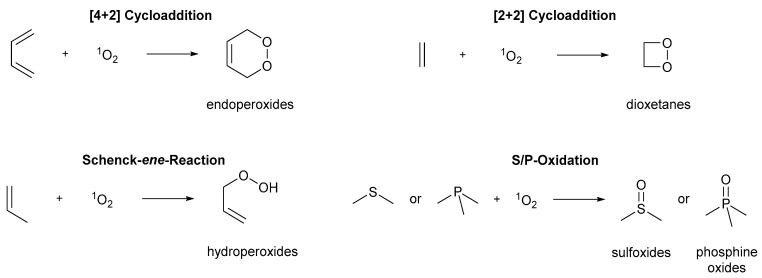
General photooxygenation reactions.

Singlet oxygen can react with a variety of functional groups (Scheme 2) [50,51,52]. Conjugated dienes preferably undergo [4+2] cycloadditions to endoperoxides, while electron-rich olefins favor [2+2] cycloadditions to 1,2-dioxetanes. Inactivated olefins with allylic hydrogen atoms undergo Schenck-*ene*-type reactions to allylic hydroperoxides instead [75,76]. Thioethers and phosphines produce the corresponding sulfoxides and phosphine oxides. All reaction types incorporate molecular oxygen into a molecular entity with high to quantitative atom economies.

Many of the industrially relevant photochemical processes convert naturally occurring or semi-synthetic starting materials to their corresponding oxygenated products [53,54,55]. Of these, essential oil derived starting materials are especially common [55,77], which makes photooxygenations promising applications for product diversification and value-adding in the essential oil industry [78]. This review will focus on demonstration (multigram)- to technical (multikilogram)-scale transformations conducted in purpose-designed solar reactors. Whenever available, key technical features of the solar reactor systems used have been provided. Related demonstration-scale photooxidations involving the super oxide anion radical (O_2_^•–^) or a thermal oxidation step were also included. In many cases, solar transformations were monitored, and obtained product mixtures analyzed by GC or GC–MS analysis techniques. Whenever necessary, the reported yields or conversions from these studies were rounded for consistency and to account for experimental errors.

## 4. Solar Preparative Photooxygenations

### 4.1. Photooxygenation of α-Terpinene and Related Reactions

**Scheme 3:**
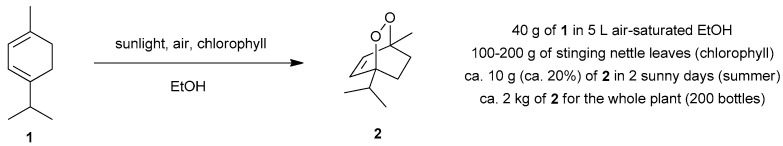
General photooxygenation reactions.

**Figure 2:**
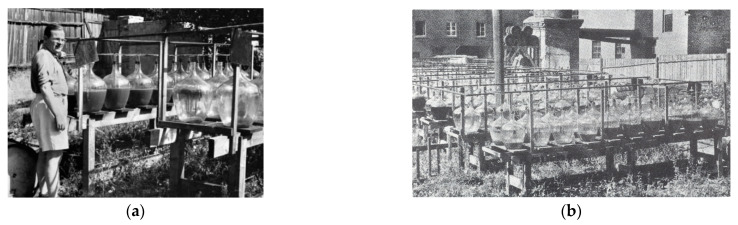
Solar production plant for the synthesis of ascaridole (**2**). (**a**) Prof. Günther Otto Schenck in front of a row of reaction bottles (taken from [47]). (**b**) The entire plant in Ziegelhausen, Germany, around 1949 (taken from [54]).

The first improvised solar manufacturing plant was built after the Second World War in Germany by the late Prof. Günther Otto Schenck (Figure 2). The plant produced ascaridole (**2**) (Scheme 3), which was urgently needed at that time to fight ascaris infections in humans [79,80,81]. The plant consisted of 200 carboy glass bottles of 10 L each that were placed in rows on wooden racks. Each flask was filled with 5 L of air-saturated ethanol solutions of α-terpinene (**1**) [54]. Stinging nettles (or spinach) leaves served as chlorophyll and hence photosensitizer source. During solar exposure, the flasks were sprayed from above with water for cooling and shaken regularly for mixing and to maintain oxygen saturation. In just two sunny summer days, the entire plant was able to produce ca. 2 kg of ascaridole (**2**). The application of ascaridole as an anthelmintic drug was later discouraged due to its severe side effects and its solar production was subsequently discontinued. Despite this, the photooxygenation of α-terpinene (**1**) still serves as a common model reaction in the development of new photosensitizers or photochemical reactor systems.

**Scheme 4:**
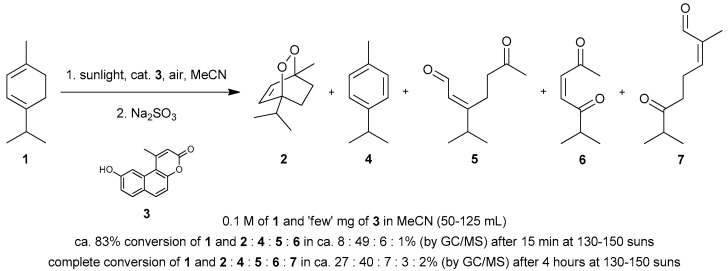
Photooxidation of α-terpinene (**1**) with benzocoumarin sensitizer **3**.

**Figure 3:**
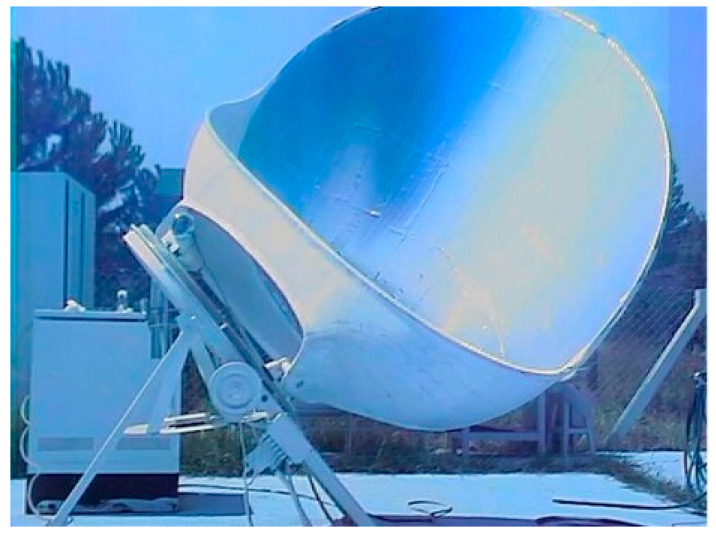
Parabolic dish solar concentrator (HTC Solar Research GmbH, Germany) at Ege University in Izmir, Turkey, around 2001 (taken from [83]).

The photooxidation of α-terpinene (**1**) in the presence of a benzocoumarin sensitizer (**3**) and in concentrated sunlight has been reported by Karapire and co-workers (Scheme 4) [82]. The authors used a small Fix Focus dish reactor with a cylindrical water-cooled Pyrex photoreactor vessel in its focal point (Figure 3). The dish concentrator had a focal length of 0.65 m, a usable reflective surface area of 2.66 m^2^ (total of 3.68 m^2^) and a reflective aluminized polymer film cover (40 μm thick layer with 0.91 g/cm^3^ of alumina). The device tracked the sun only for the elevation and required manual focusing for its azimuth. The concentration factor at the reactor’s focal point could be adjusted between 40 and 150 suns depending on experimental requirements. Solutions of **1** (0.1 M) and catalytic amounts of **3** in acetonitrile were exposed to concentrated sunlight (at 130–150 suns) for 15 min and 4 h while air was passed through the reaction mixtures. Portions of the solutions were subsequently treated with sodium sulfite (Na_2_SO_3_) and analyzed by GC–MS. Photoproducts were assigned based on their MS-spectra and characteristic molecular ions but were not isolated. Short illumination for 15 min resulted in an incomplete conversion of **1** of approx. 83% and furnished *p*-cymene (**4**) as the main product in ca. 49%. In addition, ascaridole (**2**), and the ring-opened compounds **5** and **6** were identified by GC–MS in smaller amounts of approx. 8%, 6% and 1%, respectively. In contrast, prolonged exposure to concentrated sunlight for 4 h gave complete conversion of **1** and produced *p*-cymene (**4**) and ascaridole (**2**) as the main products in amounts of ca. 40% and 27%. The ring-opened products **5**, **6** and **7** were additionally formed with approx. 7%, 3% and 2%, respectively. When the solar illumination for 4 h was repeated with nitrogen purging, no products were obtained. Based on these findings, the authors suggested that photooxidations involved both singlet oxygen (^1^O_2_) as well as super oxide anion radical (O_2_^•–^) pathways.

**Scheme 5:**
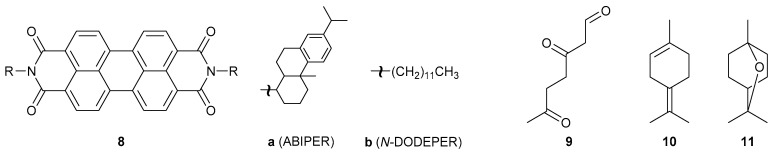
Perylenediimide sensitizers **8a** and **b** used and additional photoproducts **9-11** obtained during solar photooxidations of α-terpinene.

In an extension of this work, the same authors utilized perylenediimides as sensitizers in solution or immobilized in PVC or as sol-gel thin films on glass slides for the photooxidation of α-terpinene (**1**) (Scheme 5) [84]. Illumination experiments were conducted for 15 min and 1 h in the same small parabolic dish reactor using concentration factors of 80–90 suns (Figure 3). After treatment of the reaction mixture with Na_2_SO_3_, the products obtained were detected and assigned by GC–MS analysis. The outcome of the reactions critically depended on the photosensitizer material and solar exposure conditions. In acetonitrile solution (0.1 M of **1**) with trace amounts of ABIPER photosensitizer (**8a**), solar exposure for 1 h gave ascaridole (**2**) as the dominant photoproduct in 96%. Under the same conditions, the reaction with the **8a**-containing sol-gel thin film remained incomplete with 20% of residual **1**, and furnished ascaridole (**2**) and *p*-cymene (**4**) in amounts of ca. 49% and 18% as the main products. In contrast, the respective illumination with the PVC-immobilized sensitizer **8a** predominantly formed 3,6-dioxoheptanal (**9**) in 55%, next to 21% of ascaridole (**2**), 9% of *p*-cymene (**4**) and 16% of terpinolene (**10**), respectively. With soluble and sol-gel immobilized **8a**, shorter illuminations in concentrated sunlight for just 15 min resulted in more complex product mixtures. When *N*-DODEPER (**8b**) was used as photosensitizer in acetonitrile solution, solar exposure for 1 h resulted in approx. 70% conversion and yielded predominantly *p*-cymene (**4**) and 3-isopropyl-2-heptanal-6-one (**5**) with ca. 51% and 10%, respectively. After the same illumination time, the corresponding **8b** sol-gel thin film produced a conversion of 80% and furnished *p*-cymene (**4**) and ascaridole (**2**) as major products in ca. 34% and 15%, as determined by GC–MS. The corresponding solar reaction with **8b**-impregnated PVC generated ca. 42% of 3,6-dioxoheptanal (**9**), 30% of *p*-cymene (**4**), 11% of terpinolene (**10**) and 9% of ascaridole (**2**) as the main photoproducts instead. Shorter illuminations for 15 min again delivered different product mixtures and compositions. In the absence of any photosensitizer, GC–MS analysis after solar exposure for 1 h revealed an incomplete conversion of **1** of 57% with *p*-cymene (**4**), 3,6-dioxoheptanal (**9**), ascaridole (**2**), terpinolene (**10**) and cineol (**11**) as products in ca. 17%, 16%, 10%, 9% and 6%. In contrast, exposure to concentrated sunlight for just 15 min gave a very incomplete reaction (<7% conversion) with cineole (**11**) as the only product in approx. 7%. The formation of terpinolene (**10**) was suggested to occur via thermal rearrangement of **1**. For all other products, the authors proposed reaction pathways via the super oxide anion radical (O_2_^•–^). The variable product compositions and the small scales (25 mL) prevent any wider exploitation of this synthesis methodology.

**Scheme 6:**
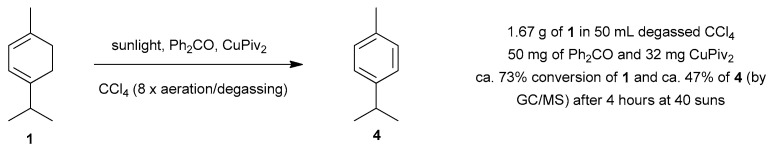
Photodehydrogenation of α-terpinene (**1**) to *p*-cymene (**4**).

The photodehydrogenation of α-terpinene (**1**) to *p*-cymene (**4**) in concentrated sunlight has likewise been reported by Avcibasi and co-workers [85]. The authors reused the small Fix Focus dish reactor shown in Figure 3 but with a concentration factor of 40 suns. An initially degassed solution of **1**, benzophenone (Ph_2_CO) and cupric pivalate (CuPiv_2_; (*^t^*BuCO_2_)_2_Cu) in carbon tetrachloride was exposed to concentrated sunlight over a period of 4 h (Scheme 6). During this exposure, the reaction was stopped eight times for aeration/degassing that oxidized cuprous (Cu^+^) back to cupric (Cu^2+^) ions. The reaction mixture was subsequently analyzed by GC–MS, which revealed a conversion of approx. 73% with ca. 47% of *p*-cymene (**4**). The postulated reaction mechanism involved hydrogen abstraction from α-terpinene (**1**) by triplet-excited benzophenone followed by reduction of the resulting radical intermediate by Cu^2+^.

### 4.2. Photooxygenation of Citronellol for the Production of Rose Oxide

**Scheme 7:**
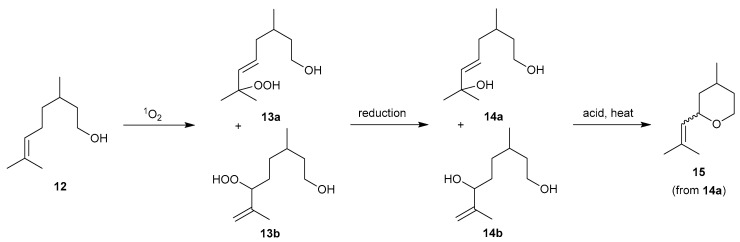
Photooxygenation of citronellol (**12**) and subsequent conversion to rose oxide (**15**).

The Schenck *ene*-photooxygenation of enantiomerically pure or racemic citronellol (**12**) is a key step in the synthesis of rose oxide (**15**), which serves as an important fragrance material in perfumery (Scheme 7) [86]. The initially obtained regioisomeric hydroperoxides **13a** and **b** are reduced to their corresponding alcohols **14a** and **b** in reported isolated yields of 60% and 35%, respectively. Subsequent acid-catalyzed rearrangement and cyclization of **14a** gives rose oxide (**15**) as a mixture of *cis*- and *trans*-diastereoisomers. Industrially, the photooxygenation of **12** is typically performed in methanol using rose bengal (RB) as a photosensitizer. After the additional thermal steps, rose oxide **15** is isolated from the crude reaction mixture by steam distillation in an overall yield of 50–60%. The annual industrial production of rose oxide has been estimated to >100 tons [87]. A modified two-phase liquid/liquid cyclization process that utilizes both regioisomers **14a** and **b** has also been patented [88].

**Figure 4:**
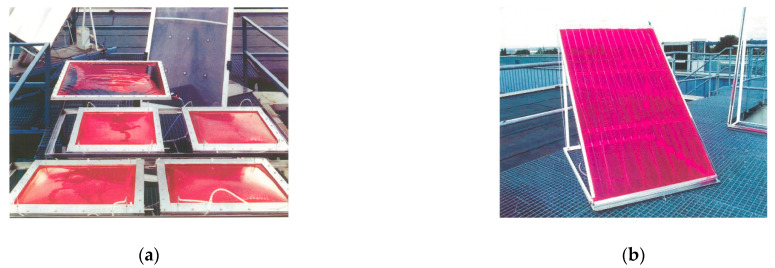
Examples of flatbed reactors developed by Prof Martin Demuth at the former Max Planck Institute for Radiation Chemistry in Mühlheim/Ruhr, Germany, between 1998–2000 (taken from [90]). (**a**) Horizonal ULCR-1T reactors. (**b**) Tilted ULCR-4P reactors.

Demuth and co-workers have developed simple flatbed reactors for solar photochemical and -thermal operations in countries with largely diffuse solar radiation [89,90]. Several models of these low-tech/low-price Universal Light Collector and Reactors (ULCR) were constructed and subsequently tested for a range of photochemical transformations. The simple ULCR-1T model (Figure 4a) was built from an aluminum frame and a reflective stainless-steel bottom plate with a Teflon^®^ cover foil (transparency >300 nm). The reactor was placed horizontally on the ground and carried inlet and outlet ports on the side of the frame, a gas inlet, a pressure release valve and optional water cooling. The more advanced and larger ULCR-4P (Figure 4b) was made from Plexiglas^®^ (poly(methyl 2-methylpropenoate), PMMA), which has a good light transparency of >300 nm. The reactor had an aperture of 1.5 m^2^ (98 W × 165 H cm), a thickness of 1.5 cm and a volume of approx. 20 L. An inlet port for gases and additional inlet and outlet ports for reagents were attached at the top of the reactor. The gas-feeding tube ran down the inside of the reactor’s body with a perforated end along its bottom. During operation, the whole device was positioned in the north–south direction and tilted to approximately 45°. The alternative ULCR-4M model (not shown) was made from more chemically inert twin-walled Makrolon^®^ (polycarbonate, PC). This material shows a lower light transparency of >400 nm, which makes it beneficial for solar transformations that do not require or are sensitive to the UV fraction of sunlight.

Selected reactor models were subsequently used for the rose bengal-sensitized photooxygenation of citronellol (**12**) in methanol (Table 1). Using the horizontal ULCR-1T reactor, solar exposure of a 6.75 M solution of **12** for 3 days under partially cloudy conditions, followed by thermal conversions, furnished rose oxide (**15**) in an isolated yield of 50% (reaction 1). In a different, not specified device made from two individual reactors connected in series, circulation of a 1.1 M solution of **12** for 11 h under mainly cloudy conditions gave complete conversion and, after further treatment, rose oxide (**15**) was isolated in 52% yield (reaction 2). For comparison, reactions 3 and 4 in a non-specified flatbed reactor were conducted in parallel in a parabolic trough reactor of a similar size (aperture of 1 m^2^) [90]. Under mainly cloudy and thus diffuse radiation conditions (reaction 3), the concentrating solar trough reactor produced a poor conversion of **12** of just about 25% after 11 h of exposure, whereas the flatbed device showed near complete consumption of citronellol (**12**). Under sunny conditions (reaction 4), both reactor systems performed equally well and effectively reached full conversions of **12** after 8 h of illumination. The latter two experiments demonstrated the superiority of the flatbed technology under non-ideal solar conditions with predominantly diffuse radiation. In all cases, the rose oxide (**15**) mixtures obtained were identical in quality to those produced from reactions using artificial light.

Technical-scale photooxygenations of citronellol (**12**) have been conducted in the advanced PROPHIS loop (Figure 1c). [59,91]. Its Helioman module comprised of four parabolic troughs equipped with Pyrex glass tubes (length 4.5 m; inner diameter 55 mm) in their focal lines. The troughs could be connected in series or in parallel. The entire concentrator had a total aperture of 32 m^2^ with 8 elements per trough. The individual mirror elements of 1 m^2^ each were made of silver-coated glass, which does not reflect any UV-light. Depending on the desired application, alternative aluminum or holographic reflectors with different reflective profiles could be fitted instead [92,93]. The reactor had a sunlight concentration factor of 30–32 suns and tracked the sun three-dimensionally with an advanced two-axis system during operation. Depending on the number of troughs employed, the operation volume ranged from 35 to 120 L. The reaction reservoir, pump, heat exchanger and gas-feeding equipment were placed in a nearby shed. Solar exposures were conducted on two different technical scales (Table 2). Isopropanol was chosen as a non-hazardous solvent and oxygen gas was injected into the reagent stream, the later circulating at 30 L/min. The first experiment (reaction 5) conducted with one trough rapidly consumed 31.8 mol of **12** within three hours of solar exposure. Subsequent reduction with aqueous sodium sulfite (Na_2_SO_3_) furnished the regioisomeric diols **14a** and **b** in a ratio of 55:45 (by GC). The second run (reaction 6) utilized all 4 troughs under optimal solar conditions and almost completely converted 43.9 mol of citronellol (**12**) within just 2¼ h. After further thermal reduction and cyclization, rose oxide (**15**) was isolated in excellent quality and yield of 55%. When the PROPHIS loop was equipped with a modified receiver tube that narrowed from 5 to 4.2 cm in inner diameter, the photooxygenation of **12** proceeded somewhat faster (reaction 7) than in the experiment with an unmodified tube (reaction 8) [94]. Although the overall reaction times were similar, the reaction conducted with the modified tube consumed 12.8 mol of **12** after one hour of illumination, compared to 10.5 mol of **12** with the standard tube. This improvement was explained by a better mixing that compensated for the depletion of oxygen along the narrowing tube.

**Figure 5:**
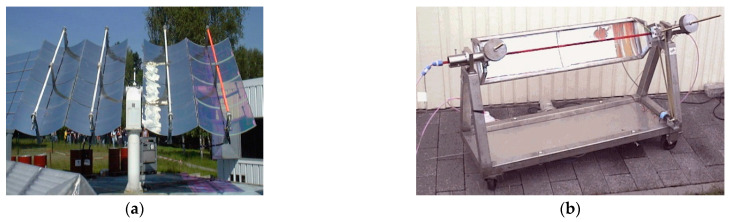
Parabolic trough concentrators equipped with holographic mirror elements at the German Aerospace Center (DLR) in Cologne-Porz, Germany. (**a**) Former PROPHIS loop, around 2002 (taken from [39]). (**b**) Custom-built laboratory reactor, around 1998 (taken from [92]).

To reduce heat-up and hence cooling-water needs, holographic mirrors were furthermore considered for solar photooxygenations [92,93]. These advanced reflectors were adapted to the sensitizer rose bengal with a reflectivity range of 480–620 nm. One trough of the PROPHIS loop was subsequently fitted with a holographic concentrator (Figure 5a). Due to difficulties with the stability of the holographic film, no experimental results have been reported publicly. Preliminary warm-up tests were, however, successfully completed in a smaller custom-built laboratory reactor with an aperture of 0.188 m^2^ (20 × 94 cm) and fitted with either aluminum or holographic elements (Figure 5b) [92]. During the experiments, a solution of rose bengal (0.4 g/L) in isopropanol was passed through the loop at a flow rate of 60 mL/min and the temperature differences between in- and outlet of the receiver tube were recorded. In comparison with the holographic mirror, the heat-up during illumination using the aluminum reflector was 60% higher due to the significant energy input of infrared radiation. Despite these encouraging results, experimental findings on the preparative photooxygenation of **12** in this device have not been published.

**Figure 6:**
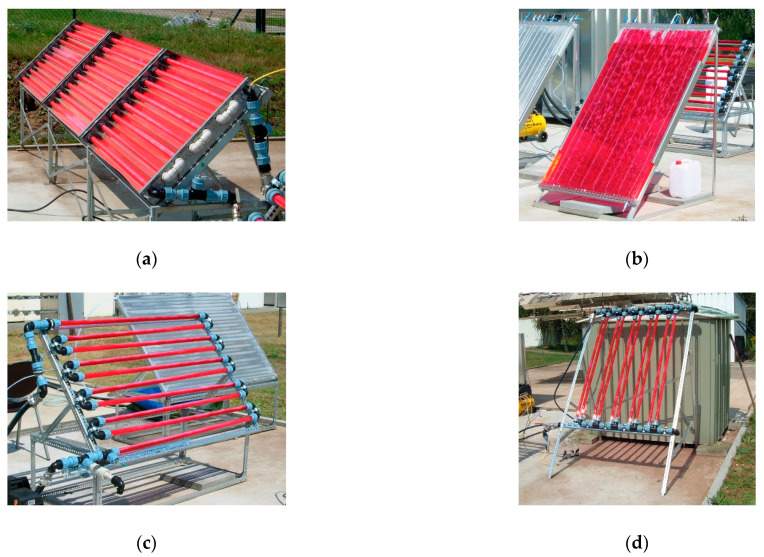
Solar reactors (except PROPHIS loop) used for photooxygenations of citronellol (**12**) at the German Aerospace Center (DLR) in Cologne-Porz, Germany in 2002. (**a**) CPC reactor. (**b**) Flatbed made from Makrolon^®^. (**c**) Horizontal serial tube reactor. (**d**) Parallel vertical tube reactor.

Due to its technical importance [54,55], the photooxygenation of citronellol (**12**) was chosen for a reactor comparison study [39,41]. Next to the advanced PROPHIS concentrator (Figure 1c), four additional non-concentrating reactor systems of different volumes, apertures, concentration factors, operation modes and oxygen feeding systems were examined (Table 3). The devices were tilted at 51° but did not follow the movement of the sun. The CPC system (Figure 6a) consisted of 8 rows of tubes that ran horizontally across three compartments and were connected in series via U-turn pipes. The flatbed reactor (Figure 6b) was an improved ULCR-4M model constructed from a triple walled Makrolon^®^ sheet. A cooling tube ran through its vertical chambers and a perforated gas-feeding tube along its bottom. The horizontal tube system (Figure 6c) was made from 10 parallel glass tubes connected in series via U-turn pipes. The vertical tube reactor (Figure 6d) was constructed from 10 glass tubes in parallel that were fed from an inlet tube on the bottom.

The horizontal and vertical tube models had no sunlight concentrator and thus solely relied on solar radiation hitting the surface of the glass tubes. Each reactor was loaded with a 10 vol% solution of **12** in isopropanol containing 0.5 g/L of rose bengal. Under ideal sunny conditions (Table 3), all reactors reached complete conversions after 2.5 h (PROPHIS) to 15 h (flatbed), respectively. Due to their superior oxygen feeding systems and hence improved oxygen saturation, the CPC and the horizontal tube device gave the highest conversion based on exposure time and illuminated area values. The PROPHIS loop showed only an average value due to its somewhat simpler oxygen supply mechanism, which only allowed gas feeding at the bottom of the first trough with relatively large oxygen bubbles formed. The poorer efficiencies of the flatbed and vertical tube reactors were explained by the inefficient gas saturation throughout these devices. Especially the compartmentalized structure of the flatbed reactor with several front and back chambers significantly limited an effective mixing and gas distribution. However, its performance could be substantially improved by operation in a circulation mode with a pump. Under less favorable cloudy conditions (Table 3), the CPC and flatbed reactors achieved complete conversions after 30 h of exposure over 4 days. In contrast, both the horizonal and the vertical tube reactors gave incomplete conversions of 66% at the end of the campaign after 33 h. The sophisticated design of the CPC concentrator and the large, fully exposed aperture of the flatbed were considered beneficial and gave superior conversions based on time and area. The small surface areas of the tube reactors naturally prevented an efficient harvesting of diffuse sunlight. The PROPHIS loop was not used under cloudy conditions due to its dependence on direct solar radiation.

An economic evaluation study for the manufacturing of rose oxide (**15**) on a 100 t/year scale in Germany has been described by Monnerie and Ortner [95]. For this, initial investment and subsequent operation costs for solar vs. lamp-driven photooxygenation processes were compared and assessed. The study suggested that the solar operation was significantly more profitable.

**Scheme 8:**
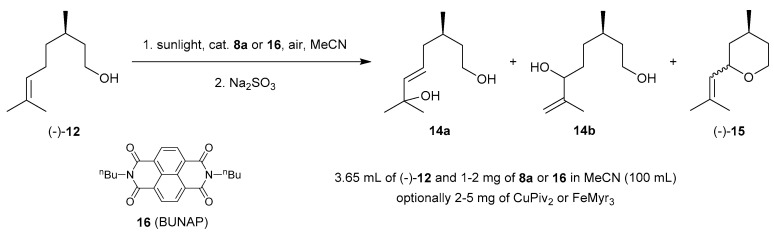
Diimide-sensitized photooxidations of (-)-**12** and subsequent conversions to rose oxide (**15**).

Dincalp and Icli investigated diimide-sensitized photooxidations of (-)-citronellol (**12**) in concentrated sunlight at 90–100 suns (Scheme 8 and Table 4) [96]. Solar exposures were performed with acetonitrile solutions of **12** (0.2 M) that were purged with air during experimental runs. Reaction mixtures were placed in a photoreactor vessel and positioned in the focal point of a dish concentrator (Figure 3). Samples were withdrawn during or after illumination and, after further thermal reduction with Na_2_SO_3_, were analyzed by GC–MS.

Using ABIPER (**8a**) as a photosensitizer (reaction 14), the conversion of (-)-citronellol (**12**) increased with prolonged illumination and after 2 h, rose oxide **15** was obtained as a diastereoisomeric mixture in a total amount of ca. 82%, next to the regioisomeric diols **14a** and **b** and residual **12** with approx. 6% and 3%, respectively. When the naphthalene diimide sensitizer BUNAP **16** was employed (reaction 15), conversions of **12** again increased with extended exposure times. The photoproduct obtained after 1 h of exposure to concentrated sunlight contained ca. 77% of rose oxide (**15**), 20% of diols **14a** and **b** and 1% of unreacted (-)-citronellol (**12**). Despite the high sunlight concentration factor, both photosensitizers remained visibly photostable throughout the experiments. In line with earlier investigations, an electron transfer mechanism involving the super oxide anion radical (O_2_^•–^) was postulated instead of a singlet oxygen (^1^O_2_) pathway. Addition of cupric pivalate (CuPiv_2_; reaction 16) or ferric myristate (FeMyr_3_; (C_13_H_27_CO_2_)_3_Fe; reaction 17) retarded the reaction and solar illuminations for 2 h reached conversions of **12** of just approximately 19% and 64%, respectively. The diasteroisomeric ratios for *cis-* and *trans-***15** varied depending on experiment, but no reasonable explanation was given to rationalize these findings.

### 4.3. Photooxygenations of β-Pinene for the Production of Myrtenol

**Scheme 9:**
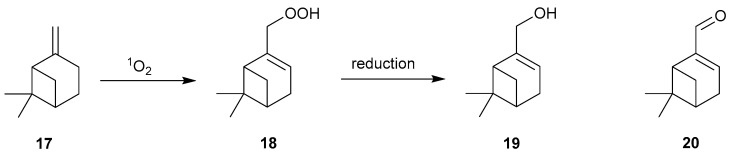
Photooxygenation of β-pinene (**17**) and subsequent conversion to myrtenol (**19**).

The Schenck *ene*-photooxygenation of β-pinene (**17**), followed by successive reduction of the hydroperoxide intermediate **18**, yields myrtenol (**19**), with myrtenal (**20**) as a common by-product (Scheme 9) [97]. Myrtenol is used as a fragrance ingredient in a variety of cosmetic, household and detergent products with an annual demand of <0.1 tons reported in 2008 [98]. Using a modified photooxygenation procedure, myrtenal (**20**) can be obtained as the main product instead [99].

Several solar photooxygenations of **17** have been independently reported and selected details are compiled in Table 5. The rose bengal-sensitized reaction of a 5 vol% solution of β-pinene (**17**) in isopropanol has been conducted at the German Aerospace Center DLR in Germany in a CPC reactor (Figure 6a). The reaction proceeded very slowly and a conversion of **17** of approximately 95% was achieved only after exhaustive exposure for 9 days (reaction 18).

**Figure 7:**
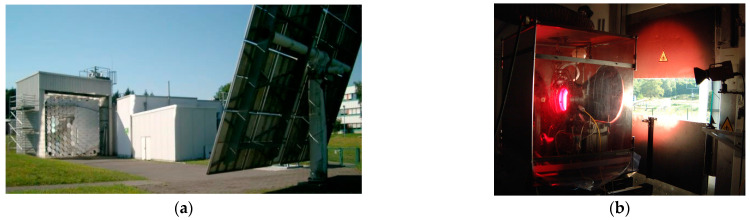
(**a**) Solar furnace at the German Aerospace Center (DLR) in Cologne-Porz, Germany, around 2005 (taken from [39]). (**b**) Custom-built photooxygenation reactor, around 2001 (taken from [39]).

To reach higher space-time yields, the reaction was transferred to the solar furnace at the DLR (Figure 7a) [100]. In this facility, sunlight is reflected by a single sun-tracking 52 m^2^ heliostat onto a concentrator made from 147 spherical mirrors (aperture of 39.1 m^2^) that directs the resulting sun beam on the experimental setup inside the laboratory room [61]. Solar photooxygenations of **17** were performed in a specialized circulating reactor fitted with an IR-filter cell and a reaction cell, the later with a total volume of approx. 5.1 L. An initial experiment was conducted in non-flammable chloroform under mainly sunny conditions using only 67 of the spherical mirrors (aperture of 18 m^2^) and tetraphenylporphyrine (TPP) as a sensitizer. Due to sensitizer bleaching and technical difficulties with the oxygen supply, the reaction was stopped after less than 3 h. After reduction with thiourea, a conversion of **17** of approx. 30% was determined by GC analysis. The solar reactor was consequently modified to allow for the usage of flammable solvents and a better oxygen feeding (Figure 7b). The improved device was then applied to rose bengal-sensitized photooxygenations of β-pinene (**17**) in ethanol. To account for possible quenching effects by breakdown products of the sensitizer rose bengal during exhaustive illumination [101], initial experiments were stopped after partial conversions of **17** of approx. 45% and 65%, respectively. After reduction with Na_2_SO_3_ and aqueous workup to remove any degradation products, the crude mixtures containing unreacted **17** and myrtenol (**19**) were subjected to further photooxygenation. Following this approach, conversions of 97% (by GC) were achieved after 14 h of exposures under largely sunny conditions. The rose bengal-sensitized photooxygenation of **17** (0.52 M) under comparable conditions without interruption and in between workup also achieved near complete conversion of 97% after 14 h of illumination (reaction 19). Quenching or retarding effects by potential photodegradation products were thus ruled out. From this experimental run, a high space-time yield of 3.6×10^-2^ mol/(L·h) was furthermore determined. However, the high construction, operation and maintenance costs of solar furnaces prevent their widespread application for preparative photochemistry.

Esser furthermore investigated the solar transformation of β-pinene (**17**) with moderately concentrated sunlight at the Plataforma Solar de Almería (PSA) in Spain in 1992 [102]. The experiment was conducted in the former SOLARIS (SOLAR photochemical synthesIS of fine chemicals) facility, an earlier version of the PROPHIS loop (Figure 1c) [103]. This parabolic trough reactor used aluminized polymeric film reflectors, had an effective concentration factor of 20 suns in practice (theoretical 42 suns) and an operating capacity of 35-70 L. Utilizing one trough (aperture of 7.29 m^2^), a solution of **17** and rose bengal (in 10 portions) in 35 L of isopropanol was passed through the loop at approx. 42 L/min while oxygen was injected into the reagent stream. The reaction temperature was maintained at roughly 20 °C and the progress of the reaction was monitored by GC analysis (reaction 20). After approximately 27 h of exposure over three consecutive days with good to excellent sunlight conditions, a conversion of β-pinene (**17**) of approx. 56% was reached, corresponding to an average conversion rate of 2.2%/h (or 0.84 mol/h). Subsequent solar thermal reduction with aqueous sodium sulfite (Na_2_SO_3_) in the SOLARIS loop furnished a crude product mixture containing ca. 40% of residual **17**, 44% of myrtenol (**19**), 7% of myrtenal (**20**) and 1% of hydroperoxide **18** (by GC), respectively.

### 4.4. Photooxygenations of α-Thujene for the Production of Trans-Sabinene Hydrate

**Scheme 10:**
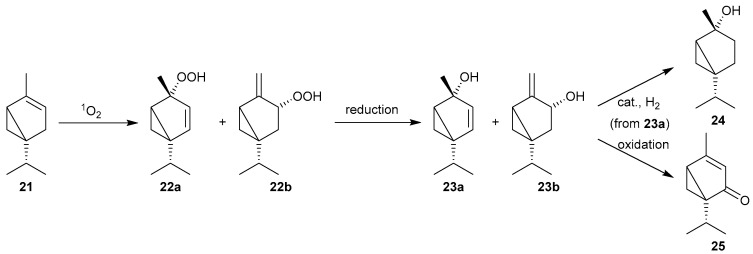
Photooxygenation of α-thujene (**21**) and subsequent conversions to *trans*-sabinene hydrate (**24**) or (-)-umbellulone (**25**).

The important and valuable flavor compound *trans*-sabinene hydrate (**24**; also known as *trans*-4-thujanol) is synthesized industrially from α- or 3-thujene (**21**) (Scheme 10) [55,104,105]. Initial photooxygenation of **21** and subsequent reduction of the regioisomeric hydroperoxides **22a** and **b** yields (-)-*trans*-4-hydroxy-β-thujene (**23a**) and (-)-*cis*-sabinol (**23b**) in a ratio of approx. 3:1. Catalytic hydrogenation of **23a** produces the desired *trans*-sabinene hydrate (**24**), while alternative oxidation furnishes the naturally occurring and somewhat toxic (-)-umbellulone (**25**).

**Figure 8:**
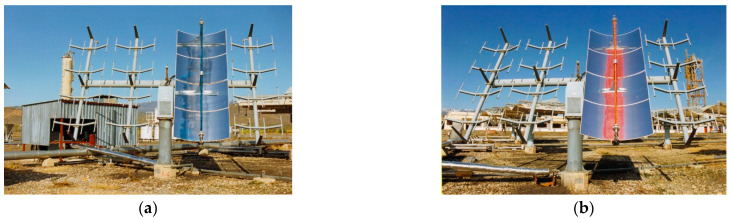
Former SOLARIS loop at the Plataforma Solar de Almería (PSA) near Tabernas, Spain, in 1992. (**a**) Used for a methylene blue and (**b**) rose bengal-sensitized photooxygenation.

In 1992, Esser conducted a series of seven solar photooxygenations of α-thujene (**21**) in isopropanol in the SOLARIS reactor equipped with one trough (Figure 8 and Table 6) [102,106]. Technical grade **21**, which contained α-pinene as impurity, with a purity of ca. 87% was employed. α-Pinene also underwent photooxygenations and its corresponding reduced photoproducts were thus detected and quantified [97]. Methylene blue (MB) or rose bengal (RB) were investigated as ^1^O_2_ photosensitizers. Both showed significant decomposition in concentrated sunlight and hence required regular refeeding during the experiments. Depending on conversion rates and weather conditions, fluid and oxygen gas flows were adjusted between 35–45 L/min and 1.0–5.5 L/min, respectively. For reactions 21–26, the reaction mixtures were cooled to approximately 20 °C during circulation within the reactor loop. Only for reaction 27, the temperature was increased to approx. 30 °C instead. Based on the total solar radiation recorded, the corresponding moles of photons received in the absorption range of the respective sensitizer, and hence solar photon yields (η_s_) were calculated. All solar photooxygenations proceeded efficiently and furnished high to near complete conversions of **21** of >87% after exposure times of 4½-11 h. Due to the instability of the hydroperoxide intermediates **22a** and **b**, their subsequent solar thermal reductions were directly performed inside the SOLARIS loop at temperatures >50 °C. Except for reaction 22, high selectivities for (-)-*trans*-4-hydroxy-β-thujene (**23a**) formation of ≥72% were achieved. The best overall results with respect to exposure time, conversion, selectivity and solar photon yield were accomplished with rose bengal at 20 °C (reactions 25 and 26). From the combined crude product mixture of all experiments, the desired (-)-*trans*-4-hydroxy-β-thujene (**23a**) was isolated in 78% purity (by GC) and 65% yield, both exceeding the reported values for lamp-driven processes. This was explained by the smaller UV-content of the reflected natural sunlight.

### 4.5. Photooxygenations of Furfural and Furfuraldiethylacetal to 5-Hydroxy- and 5-Alkoxyfuranones

**Scheme 11:**
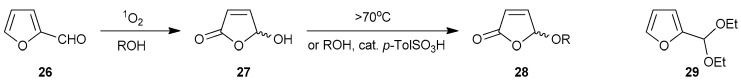
Photooxygenation of furfural (**26**) to 5-hydroxyfuranone (**27**) and subsequent conversion to 5-alkoxyfuranones (**28**).

The photooxygenation of furfural (**26**) in various alcohols was first described by Schenck, who isolated the corresponding 5-alkoxyfuranone derivatives **28** (Scheme 11) [106,107]. It was later shown that 5-hydroxyfuranone (**27**) is formed as the initial photoproduct, which can be subsequently isolated in yields of ≤90% [108,109]. The pseudo-acid **27** is rapidly converted to **28** at elevated temperatures >70 °C, which were commonly exceeded in earlier studies. Hydroxyfuranone (**27**) has pesticidal properties but also serves as a versatile C_4_ building block in organic synthesis [110]. Longer-chain 5-alkoxyfuranones, prepared by acid-catalyzed (trans)esterification, have been described as fragrances [111].

The solar exposure of a solution of **26** (100 g) and eosin (1 g) in ethanol (1 L) in a large conical flask has been described in a patent in 1953 [108]. After 1½ days in direct sunlight, 5-ethoxyfuranone (**28**; R = Et) was isolated in a yield of 50% (66.7 g).

In 1991 and 1992, sixteen large-scale photooxygenations were subsequently investigated in the SOLARIS loop equipped with a single trough (Figure 8) [47,102]. Process conditions were systematically varied and included alterations in the starting material (**26** vs. **29**), photosensitizer (RB vs. MB), temperature, fluid flow rates and possible additives. Five selected examples from this solar campaign are summarized in Table 7 [47]. The oxygen gas flow rates were adjusted based on the weather conditions and reaction progress and ranged between 0.7 and 5.5 L/min. Methylene blue was identified as the preferred photosensitizer due to its superior stability under the weakly acidic reaction conditions. Despite varying weather conditions, all solar reactions furnished near complete conversions of >95% after 3¼-16 h of illumination. The reaction was found to be very robust and partially converted reaction mixtures could be kept in the reactor loop during bad weather periods. The addition of concentrated hydrochloric acid (reaction 31) was found beneficial due to the rapid formation of furfuraldiethylacetal (**29**), which reacted more rapidly with singlet oxygen than furfural (**26**) itself. This was demonstrated when diethylacetal **29** was used as the starting material (reaction 32). While the selectivity for the formation of hydroxyfuranone (**27**) was high during the actual photooxygenation, significant conversion into ethoxyfuranone (**28**; R = Et) occurred during further workup and isolation. No attempts to isolate the more desirable **27** from these mixtures were consequently made. However, the acid-catalyzed (trans)esterification of a crude mixture (ca. 250 g) containing **27** and **28** (R = Et) with *n*-hexanol to hexyloxyfuranone (**28**; R = Hex) in 79% yield has been reported in a patent [111].

### 4.6. Photooxygenations of 1,5-Dihydroxynaphthalene to Juglone

**Scheme 12:**
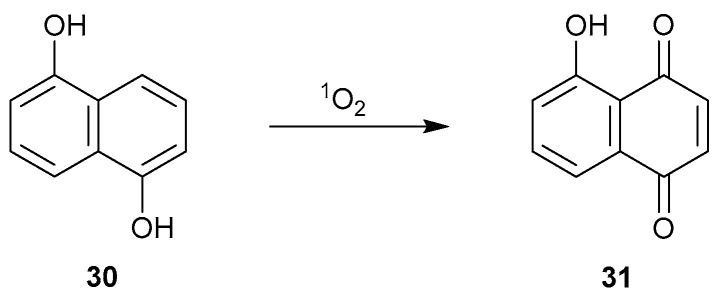
Photooxygenation of 1,5-dihydroxynaphthalene (**30**) to juglone (**31**).

Juglone or 5-hydroxy-1,4-naphthoquinone (**31**) is a naturally occurring compound that is used as a herbicide, dye, coloring agent and versatile building block chemical [112]. It can be obtained in good yields from 1,5-dihydroxynaphthalene (**30**) through photooxygenation (Scheme 12) [113].

**Figure 9:**
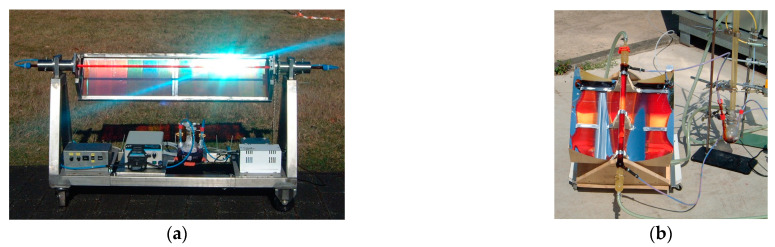
Laboratory-scale parabolic trough concentrators at the German Aerospace Center (DLR) in Cologne-Porz, Germany. (**a**) Horizontal trough reactor equipped with holographic mirrors (the reflected green light can be clearly seen), 2003. (**b**) Vertical trough reactor based on a Liebig condenser, 2003 (taken from [115]).

Small laboratory-scale photooxygenations of **30** (0.5-2 g) have been conducted by Oelgemöller and co-workers in custom-built parabolic trough reactors (Table 8). Initial experiments were performed in a horizontal trough reactor (Pyrex tube diameter of 1.2 cm) equipped with holographic mirror elements and using isopropanol as a solvent (Figure 9a) [91]. The reactor had an optimal reflectivity range for the usage of rose bengal of 480–620 nm [92,93], aperture of 0.188 m^2^ and a concentration factor of approx. 15 suns. Oxygen gas was injected into the circulating reagent flow in a Y-connector, thus generating an uneven gas-liquid flow. Conversion rates of ≥83% were achieved after illuminations for 3 h and 8 h and juglone (**31**) was isolated in yields of 54% and 79%, respectively (reactions 33 and 34).

Using the identical reactor equipped with a polished aluminum reflector instead, the same authors tested different solvents (isopropanol vs. acetone) and sensitizer materials (RB vs. MB; soluble vs. solid-supported) [114]. Applying a standard solution of 2 g of diol **30** in 250 mL of solvent and a fixed exposure time of four hours, the experiments conducted in either solvent with soluble rose bengal showed the best performances (reactions 35 and 36). Both runs achieved high conversions of ≥93% and furnished juglone (**31**) in isolated yields of 75% and 79%, respectively. The solid-supported sensitizing materials gave significantly lower conversion rates of 46-70% after the same illumination time, presumably due to the poor transport and thus mixing of these materials within the reactor’s tube.

Three additional solar photooxygenations involving diol **30** were furthermore conducted in a simple vertical parabolic trough reactor that used a Liebig condenser with a diameter of 2.4 cm as receiver tube (Figure 9b) [115]. The device used a bent polished aluminum sheet with an aperture of approx. 0.15 m^2^ (41 × 36 cm) and had a theoretical concentration factor of ca. 18 suns. The reaction mixture containing **30** (0.5-1.0 g) in either isopropanol or methanol (100 mL) was pumped through the outer mantle of the condenser, while cooling water was passed upwards through its central tube. After solar illuminations for effectively ^2^/_3_–4½ h, conversion rates of **30** of ≥86% and isolated yields of juglone (**31**) of 46–71% were obtained. The highest conversion and isolated yield were again achieved using rose bengal and isopropanol (reaction 37).

### 4.7. Miscellaneous Solar Photooxygenations and Photooxidations

**Scheme 13:**
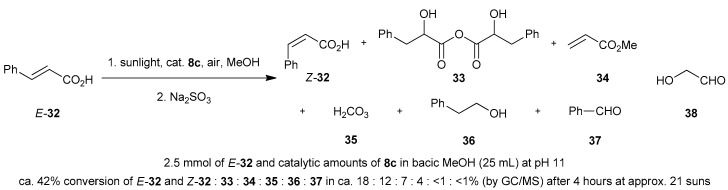
SULFAPER-sensitized photooxidations of *E*-cinnamic acid (*E-***32**).

Sensitized photooxidations of *E*-cinnamic acid (*E*-**32**) and methyl acrylate (**34**) in a parabolic dish concentrator (Figure 3) have been described by Dincalb and Icli [116]. Aerated solutions of *E*-**32** or **34** in methanol and in the presence of sulfoamino perylenediimide **8c** (SULFAPER; R = -(CH_2_)_12_NHSO_2_OH) were exposed to concentrated sunlight (approximately 21 suns) for up to four hours and after successive reduction, the product mixtures were analyzed by GC–MS coupling. Illumination of *E*-**32** for four hours (Scheme 13) showed incomplete conversion and furnished a complex reaction mixture with *Z*-cinnamic acid (*Z*-**32**) and the anhydride **33** as the main products in amounts of ca. 18% and 12%, respectively. Methyl acrylate (**34**), carbonic acid (**35**), 2-phenylethanol (**36**) and benzaldehyde (**37**) were identified as minor products. Additional exposure in the absence of **8c** and under deaerated or aerated conditions delivered somewhat different product mixtures and 2-hydroxyacetaldehyde (**38**) was furthermore detected. In all case examined, direct photoisomerization to the isomeric *Z*-cinnamic acid (*Z*-**32**) was found as the dominate reaction pathway. The formation of **33**, **36** and **37** was explained via photoinduced electron transfer and the involvement of the super oxide anion radical (O_2_^•–^). The small scale and the rather complex outcome of this transformation limit its usefulness in organic synthesis.

**Scheme 14:**
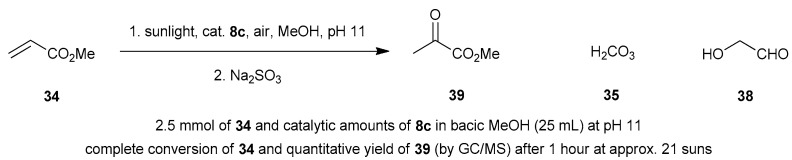
SULFAPER-sensitized photooxidations of methyl acrylate (**34**).

The SULFAPER-sensitized reaction of methyl acrylate (**34**) in basic methanol was much more selective (Scheme 14) [116]. Exposure to concentrated sunlight under aerated conditions for one hour furnished complete conversion of **34** and methyl 2-oxopropanoate (**39**) was obtained as the only product. Shorter illumination for ¼ hour under the same conditions also produced carbonic acid (**35**) in ca. 10%, next to **39** and residual **34** in amounts of approximately 69% and 21%, respectively. In the absence of the photosensitizer **8c**, solar exposure for one hour resulted in a low conversion of just ca. 27% and gave carbonic acid (**35**) and 2-hydroxyacetaldehyde (**38**) as the only photoproducts in amounts of ca. 16% and 11%. Under deareated conditions in the presence of SULFAPER, illuminations for ¼ or one hour yielded methyl 2-oxopropanoate (**39**) as the sole product in amounts of approx. 57% and 68% next to residual **34**. Electron transfer steps including the generation of the super oxide anion radical (O_2_^•–^) were postulated to explain the formation of **39**. Despite the selective nature of this particular transformation, the rather demanding technical requirements and the small reaction scale hinder a wider application.

**Scheme 15:**
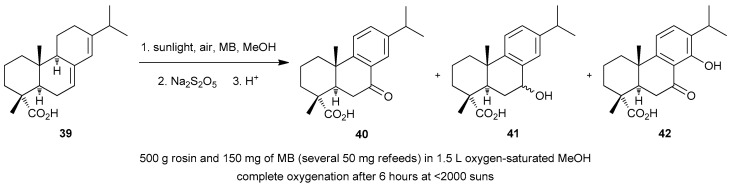
Photooxygenation and thermal reduction of abietic acid (**39**), the major component in rosin, to its oxygenated products **40**–**42**.

The solar photooxygenation of natural rosin or collophany, which is obtained from pine trees, has been investigated by Icli [117]. A solution of rosin in methanol containing methylene blue was exposed to concentrated sunlight in a Bomin Solar fixed focus concentrator capable of concentration factors of up to 2000 suns. A circulating reactor was positioned in the focal point (1–2 cm in diameter) of the solar dish. The reactor consisted of a double-jacked Pyrex tubing of 30 cm length with an outer diameter of 5.5 cm and an inner diameter of 4 cm. This reaction tube was connected to a 2 L reservoir flask, which was cooled in an ice bath during operation. The reaction mixture was purged with oxygen in the reservoir and then circulated through the inner reaction tubing at 500 mL/min, while colling water was passed through the outer mantle. After six hours of circulation with occasional refeeding of methylene blue, the reaction product was treated with sodium metabisulfite (Na_2_S_2_O_5_), acidified to pH 4–5 and the precipitated oxygenated rosin collected and analyzed. NMR and IR analyses revealed the presence of photooxygenated products with potentially enhanced emulsifier properties. As rosin consists mainly of abietic acid (**39**), its photooxygenation-reduction sequence to the oxygenated products **40**–**42** is exemplarily shown in Scheme 15. The author also suggested a potential industrial production of photooxygenated rosin from agricultural leftover stocks using four parabolic trough collectors with a moderate concentration factor of 50–100 suns and an aperture of 10 m^2^ each connected in series. In favorable regions with high levels of solar radiation, the plant was estimated to convert 100 kg of rosin in 300 L of methanol in three hours in summer, four to five hours in spring and six to eight hours in winter, respectively.

**Scheme 16:**
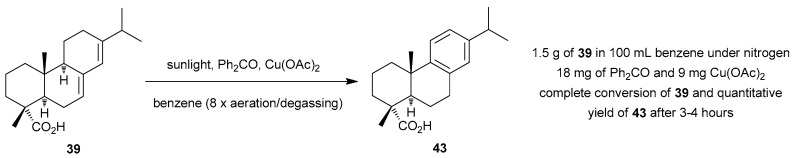
Photodehydrogenation of abietic acid (**39**) to dehydroabietic acid (**43**).

Sensitized dehydrogenation reactions of natural pine rosin and abietic acid (**39**) were furthermore studied by Icli and co-workers in concentrated sunlight (Scheme 16) [118]. The authors utilized a non-specified solar dish concentrator with a diameter of 1 m and a cooled tube-shaped reactor placed in its focal point. Solutions containing rosin or **39**, benzophenone (Ph_2_CO) and copper acetate (Cu(OAc)_2_; (CH_3_CO_2_)_2_Cu) in benzene were exposed to sunlight under nitrogen for up to three hours. Each experimental run was stopped eight times for aeration/degassing to regenerate cupric (Cu^2+^) ions. Following this approach, abietic acid (**39**) was quantitatively converted to dehydroabietic acid (**43**). The authors concluded that the selective solar dehydrogenation to **43** may have commercial potential, although the reaction scale and hazardous solvent necessitate further optimization studies.

**Scheme 17:**
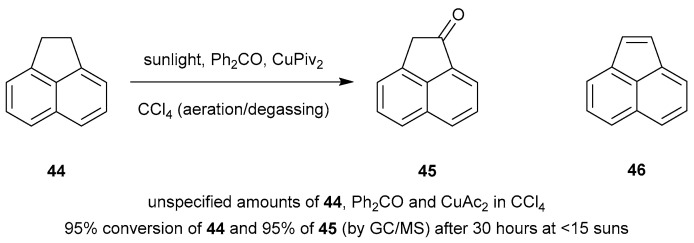
Photodehydrogenation of acenaphthene (**44**) to acenaphthenone (**45**).

The benzophenone-sensitized photodehydrogenation of acenaphthene (**44**) in the presence of cupric pivalate (CuPiv_2_) was only observed in concentrated sunlight [85]. Using a fix focus solar dish reactor with a sunlight concentration factor below 15 suns (Figure 3), a degassed solution of **44**, benzophenone and CuPiv_2_ in carbon tetrachloride was illuminated for 30 h over a seven-day period with several aeration/degassing cycles. Subsequent GC–MS analysis revealed the clean formation of acenaphthenone (**45**) in 95%, next to 5% of unreacted **44** (Scheme 17). Continuous solar exposure under deaerated conditions for 7½ h with a concentration factor of under 110 suns resulted in a low conversion of just 3% with acetnaphthylene (**46**) detected by GC–MS in an amount of <1%. Prolonged illuminations for up to 20 h under the same conditions somewhat increased conversion rates to 11%, but with only ca. 2% of **46** formed. The latter experiments unambiguously showed the importance of molecular oxygen (^3^O_2_) for catalyst regeneration.

Icli and co-workers also investigated the naphthalene diimide-sensitized photooxidation of anthracene to its corresponding endoperoxide in the HTC reactor under 10.2 suns (Figure 3) [119]. However, no further experimental details were provided by the authors.

## 5. Limitations, Challenges and Opportunities

Although photooxygenations have been successfully realized ‘outdoors’ in natural sunlight on the demonstration to technical scales, many reported processes suffer from experimental or technical drawbacks. For example, very few solar manufacturing studies, notably rose oxide (**15**) [59], (-)-*trans*-4-hydroxy-β-thujene (**23a**) [102] and juglone (**31**) [91,114,115], have truly isolated the oxygenated target products of interest. Most investigations obtained crude products instead and determined compositions and ‘yields’ simply by NMR, GC or GC–MS analyses. A further conversion of a crude reaction mixture followed by isolation of the desired product (hexyloxyfuranone **28**; R = Hex) has nevertheless been described [111]. Some processes generated complex mixtures, hence requiring time and resource demanding separation and purification steps. Several protocols also involved hazardous and flammable solvents from the corresponding ‘indoor’ (laboratory) procedure, which need major risk control measures. Other studies utilized highly sunlight-concentrating facilities such as parabolic dish systems or solar furnaces that place the reaction chamber within their focal points. Next to the high installation costs, these concentrators generate large amounts of heat and thus demand sophisticated and reliable cooling systems for effective heat management. Due to the limited capacity of the actual reaction chambers, these systems are also difficult to scale to industrially relevant productivities.

Preparative solar chemistry has seen a surge between 1990 and 2005, largely driven by dedicated research programs involving the PSA in Spain and the DLR in Germany [120]. Despite the realization of a variety of attractive solar transformations during this period, the developed technologies and processes were not implemented by the chemical industry. A major hurdle for adaptation may have been the high initial investment costs, especially for more advanced sunlight concentrator systems, and the need for ‘operation security’ independent of location or weather. In contrast to the more complex trough or dish reactors, low sunlight-concentrating systems such as the CPC reactor are easy to manufacture and maintain and can furthermore utilize direct and diffuse radiation. This makes this device advantageous for the construction of solar chemical plants, as already successfully demonstrated for large-scale wastewater treatment processes [57,58]. The initial construction costs for these facilities may be offset by annual savings on energy, cooling water and replacement lamps [95]. At present, the transfer of existing photochemical processes from the fragrance and flavor industry to solar operations appears economically and technically viable. These (and other) fine and specialty products are manufactured on scales below 1000 tons/year and thus do not necessarily require continuous, year-round operation [121,122,123].

Naturally, the location of any future synthetic-organic solar industry will demand favorable sunlight conditions, but also existing infrastructure and access to target markets. For larger chemical markets or less favorable locations, however, tandem solar lamp-driven processes can overcome the reliance on the day–night cycle and hence guarantee a continuous production of target materials. Under inadequate sunlight conditions or at night, energy efficient light sources such as LEDs or OLEDs can drive or support the photochemical process [13,124,125]. Prototype solar lamp-driven devices have indeed already been developed and applied for water treatment applications [126,127]. Solar photochemistry also offers economic benefits for decentralized manufacturing, for example small-scale conversions of local biomass in the (tropical) essential oil industry or in developing countries [128,129]. In isolated and remote locations, photovoltaic (PV) panels can provide power for electrical components, as again successfully demonstrated for water treatment applications [130,131].

## 6. Conclusions and Outlook

Photooxygenations are ideal candidates for preparative solar applications with significant future synthesis potential [132,133]. Industrially relevant and new processes have been realized using a variety of solar reactor systems, clearly demonstrating the capability of this methodology. Current or future technologies and processes developed can be further adopted and transferred to other photochemical or even thermal applications [39,40,41,42,43,44,45,46,47,134]. Visible-light photoredox catalysis, for example, has seen remarkable growth over the last decade [135,136,137], but no large-scale solar processes have been reported yet [138]. Likewise, new solar chemical reactor concepts have been recently developed, including continuous-flow trough and luminescent solar concentrators [139,140,141]. These chemical and technological advances have complemented past and existing activities in green photochemistry [142,143,144,145,146,147,148]. It is hoped that the future of solar photochemistry in general as a sustainable synthesis tool will be bright [149].

## Figures and Tables

**Table 1:** Experimental details of photooxygenations of citronellol (**12**) conducted in flatbed reactors.

	Reaction 1 [89]	Reaction 2 [89]	Reaction 3 [90]	Reaction 4 [90]
citronellol (**12**)	0.5 L (2.7 mol)	0.2 L (1.1 mol)	0.1 L (0.55 mol)	0.1 L (0.55 mol)
methanol	400 mL	1 L	2 L	2 L
rose bengal ^1^	5 × 6 g	3 g + 1 g	5 g	5 g
reactor	batch (1 m^2^)	in series (1 m^2^)	batch (1 m^2^)	batch (1 m^2^)
operation	static	flow (2 L/h)	static	static
time	3 days	11 h	11 h	7 h
solar conditions	50% cloudy, 50% sunny	70% cloudy; 30% sunny	70% cloudy; 30% sunny	100% sunny
yield of **15** ^2^	50% (1.36 mol)	52% (0.57 mol)	not reported ^3^	not reported ^3^

^1^ Added in several dosages. ^2^ Isolated yield based on **12** after further reduction and cyclization. ^3^ >95% conversion of **12**.

**Table 2:** Experimental details of photooxygenations of **12** conducted in the PROPHIS loop.

	Reaction 5 [91]	Reaction 6 [91]	Reaction 7 [94]	Reaction 8 [94]
citronellol (**12**)	5.8 L (31.8 mol)	8 L (43.9 mol)	5.8 L (31.8 mol)	5.8 L (31.8 mol)
isopropanol	40 L	72 L	40 L	40 L
rose bengal	20 g ^1^	36 g ^1^	2 g	2 g
O_2_-flow	600 L/h	200 L/h	not reported	not reported
# of troughs	1 (8 m^2^)	4 (32 m^2^)	1 (8 m^2^) ^2^	1 (8 m^2^)
time ^3^	3 h	2¼ h	2⅓ h	2¼ h
solar conditions	largely sunny	sunny	sunny	sunny
conversion of **12** ^4^	>95%	>95% (55% ^5^)	>95%	>95%

^1^ Added in several dosages. ^2^ Modified tube with narrowing diameter. ^3^ Time until maximum conversion was reached. ^4^ Determined by GC (vs. internal standard) after reduction with Na_2_SO_3_. ^5^ Isolated yield of **15** based on **12** after further reduction and cyclization.

**Table 3:** Reactor and experimental details of the solar campaign.

	Reactions 9	Reactions 10	Reactions 11	Reactions 12	Reactions 13
reactor	PROPHIS	CPC	flatbed	horizontal tubes	vertical tubes
CF	32 suns	1 sun	1 sun	1 sun	1 sun
aperture	32 m^2^	3 m^2^	1.5 m^2^	not given	not given
volume	80 L (37 L ^1^)	40 L (26 L ^1^)	21 L ^1^	25 L (11 L ^1^)	20 L (10 L ^1^)
operation	circulation	circulation	static	circulation	circulation
mixing	static mixer	7 × 180° turns	air bubbling	9 × 180° turns	air bubbling
citronellol (**12**)	8 L (43.9 mol)	4 L (22 mol)	2.1 L (11.5 mol)	2.5 L (13.7 mol)	2 L (11 mol)
isopropanol	72 L	36 L	18.9 L	22.5 L	18 L
rose bengal ^2^	40 g	20 g	10.5 g	12.5 g	10 g
O_2_ source	oxygen	air	air	air	air
time (sunny/cloudy) ^4^	2½ h ^3^/not operated	7 h/30 h	15 h/30 h	12 h/33 h	13 h/33 h
conversion of **12** ^5^	>95% ^3^/not operated	>95%/>95%	>95%/>95%	>95%/>65%	>95%/>65%

^1^ Exposed volumes (all tubes). ^2^ Added in several dosages. ^3^ Reaction 6 from Table 2. ^4^ Illumination time under sunny/cloudy conditions. ^5^ Determined by GC (vs. internal standard) after subsequent reduction with Na_2_SO_3_.

**Table 4:** Experimental details of diimide-sensitized photooxidations of (-)-**12**.

	Reaction 14	Reaction 15	Reaction 16	Reaction 17
sensitizer	ABIPER (**8a**)	BUNAP (**16**)	ABIPER (**8a**)	ABIPER (**8a**)
additive	none	none	CuPiv_2_	FeMyr_3_
exposure time ^1^	2 h	1 h	2 h	2 h
residual **12** ^2^	3%	1%	81%	36%
**14a**/**14b**^2^	6%	20%	2%	5%
**15** (*cis*/*trans* ^3^) ^2^	76%/6%	54%/23%	11%/5%	53%/6%

^1^ Maximum exposure times. ^2^ Determined by GC after subsequent reduction with Na_2_SO_3_. ^3^ Diasteroisomers.

**Table 5:** Reactor and experimental details of selected solar photooxygenations of β-pinene (**17**).

	Reaction 18	Reaction 19 [100]	Reaction 20 [102]
reactor	CPC	DLR solar furnace	SOLARIS
CF	1 sun	up to 4,800 suns	20 suns (42 suns ^1^)
volume	40 L (26 L ^2^)	5.1 L (2.03 L ^2^)	70 L (30 L ^2^)
β-pinene (**17**)	2 L (12.8 mol)	0.29 L (1.84 mol)	6.2 L (37.7 mol)
solvent	38 L (^i^PrOH)	3.5 L (EtOH)	35 L (^i^PrOH)
rose bengal	not reported	40 g ^3^	21.77 g ^3^
exposure time	9 days	14 h	3 days (ca. 27 h)
conversion of **17** ^4^	95%	97%	ca. 56%

^1^ Theoretical concentration factor without optical losses. ^2^ Exposed volumes of reactor. ^3^ Added in several dosages. ^4^ Determined by GC directly or after subsequent reduction with Na_2_SO_3_.

**Table 6:** Experimental details of photooxidations of **21**.

	Reaction 21	Reaction 22	Reaction 23	Reaction 24	Reaction 25	Reaction 26	Reaction 27
α-thujene (**21**) ^1^	2.7 kg (17.2 mol)	5.42 kg (34.5 mol)	5.45 kg (34.6 mol)	~3.3 kg (20.9 mol)	5.63 kg (35.8 mol)	5.64 kg (35.9 mol)	5.63 kg (35.8 mol)
^i^PrOH	44 L	38 L	35 L	~32 L	35 L	35 L	35 L
sensitizer ^2^	5.26 g (MB)	9.61 g (MB)	11.11 g (MB)	12.29 g (MB)	12.82 g (RB)	9.02 g (RB)	6.95 g (RB)
time ^3^	~7 h (1 day)	~11 h (2 days)	7 h (1 day)	~7 h (1 day)	6 h (1 day)	5½ h (1 day)	4½ h (1 day)
total radiation ^4^	16.5 kWh (110 mol)	37.9 kWh (271 mol)	40.8 kWh (287 mol)	43.0 kWh (306 mol)	29.9 kWh (123 mol)	27.5 kWh (113 mol)	24.2 kWh (106 mol)
η_s_^5^	14%	12%	12%	7%	29%	31%	33%
conversion ^6^	88%	97%	96%	97%	~100%	99%	98%
**21** ^7^	12%	2%	3%	2%	<1%	1%	1%
**23a** ^7^	61%	56%	63%	66%	65%	67%	61%
**23b** ^7^	7%	11%	11%	10%	10%	11%	13%
selectivity ^8^	82%	67%	75%	78%	75%	78%	72%

^1^ Technical grade (86.5%). ^2^ Sensitizer added in several dosages. ^3^ Illumination time (over number of days). ^4^ Calculated molar radiation up to 700 nm (MB) or 600 nm (RB). ^5^ Solar photon yield. ^6^ Conversion of **21** by GC. ^7^ Product composition by GC after reduction with Na_2_SO_3_ (by-products excluded). ^8^ Selectivity for **23a**.

**Table 7:** Experimental details of selected photooxidations of furane derivatives **26** and **29**.

	Reaction 28	Reaction 29	Reaction 30	Reaction 31	Reaction 32
furane	2.06 kg 21.5 mol (**26**)	2.14 kg 22.3 mol (**26**)	4.32 kg 45.0 mol (**26**)	3.61 kg 37.6 mol (**26**)	3.61 kg 21.2 mol (**29**)
ethanol	35 L	35	30	35	35
sensitizer ^1^	6.1 g (MB)	11.2 g (RB)	5.1 g (MB)	9.0 g (MB)	6.0 g (MB)
additive	none	none	none	c. HCl (5 mL)	none
temperature	8 °C	20 °C	20 °C	20 °C	20 °C
fluid flow	35 L/min	55 L/min	55 L/min	45 L/min	45 L/min
time ^2^	16 h (3 days)	~11 h (2 days)	~12½ h (2 days)	5½ h (1 day)	~3¼ h (1 day)
total radiation ^3^	22.2 kWh 140 mol	54.3 kWh 413 mol	61.9 kWh 436 mol	32.4 kWh 226 mol	16.9 kWh 114 mol
η_s_^4^	15%	5%	10%	16%	18%
conversion ^5^	98%	99%	~100%	98%	95%
**27**:**28**^6^	42:1	133:1	58:1	98:1	390:1

^1^ Sensitizer added in several dosages. ^2^ Illumination time (over number of days). ^3^ Calculated molar radiation up to 700 nm. ^4^ Solar photon yield. ^5^ Conversion of **26** or **29** by GC. ^6^ Ratio of **27** vs. **28** (R = Et) by GC.

**Table 8:** Experimental details of selected photooxidations of 1,5-dihydroxynaphthalene (**30**).

	Reaction 33	Reaction 34	Reaction 35	Reaction 36	Reaction 37
reactor	horizontal trough	horizontal trough	horizontal trough	horizontal trough	vertical trough
CF	15 suns	15 suns	15 suns	15 suns	18 suns
reflector	holographic	holographic	aluminum	aluminum	aluminum
diol **30**	2.0 g	1.0 g	2.0 g	2.0 g	0.5 g
solvent	200 mL (^i^PrOH)	200 mL (^i^PrOH)	250 mL (^i^PrOH)	250 mL (acetone)	100 mL (^i^PrOH)
sensitizer	0.1 g (RB)	0.1 g (RB)	0.1 g (RB)	0.1 g (RB)	0.05 g (RB)
time	8 h ^1^ (2 days)	3 h ^1^ (2 days)	4 h ^1^ (1 day)	4 h ^1^ (1 day)	^2^/_3_ h ^1^ (1 day)
conversion ^2^	83%	>95%	93%	99%	>95%
yield of **31** ^3^	54%	79%	75%	79%	71%

^1^ Illumination time (over number of days). ^2^ Conversion of **30** by GC. ^3^ Isolated yields of **31**.
